# Lipoprotein(a) and its role in inflammation, atherosclerosis and malignancies

**DOI:** 10.1007/s11789-017-0084-1

**Published:** 2017-02-10

**Authors:** Evelyn Orsó, Gerd Schmitz

**Affiliations:** Institute for Clinical Chemistry and Laboratory Medicine, University Hospital of Regensburg, Franz-Josef-Strauss-Allee 11, 93053 Regensburg, Germany

**Keywords:** Lipoprotein(a), Pathophysiology, Atherogenesis, Inflammation, Tumor-growth

## Abstract

Lipoprotein (a) (Lp(a)) is a modified low-density lipoprotein (LDL) particle with an additional specific apolipoprotein (a), covalently attached to apolipoprotein B‑100 of LDL by a single thioester bond. Increased plasma Lp(a) level is a genetically determined, independent, causal risk factor for cardiovascular disease.

The precise quantification of Lp(a) in plasma is still hampered by mass-sensitive assays, large particle variation, poor standardization and lack of assay comparability.

The physiological functions of Lp(a) include wound healing, promoting tissue repair and vascular remodeling. Similarly to other lipoproteins, Lp(a) is also susceptible for oxidative modifications, leading to extensive formation of pro-inflammatory and pro-atherogenic oxidized phospholipids, oxysterols, oxidized lipid-protein adducts in Lp(a) particles, that perpetuate atherosclerotic lesion progression and intima-media thickening through induction of M1-macrophages, inflammation, autoimmunity and apoptosis. The oxidation-specific epitopes of modified lipoproteins are major targets of pre-immune, natural IgM antibodies, that may attenuate the pro-inflammatory and pro-atherogenic effects of Lp(a).

Although the data are still insufficient, recent studies suggest a potential anti-neoplastic role of Lp(a).

## Introduction

Lipoprotein(a) (Lp(a)) is one of the most enigmatic lipoprotein particles in humans and its increased plasma concentration is an independent predictor for atherosclerotic cardiovascular disease (CVD) and peripheral arterial disease ([1] and references therein). Mendelian randomization studies have recently provided convincing evidence that Lp(a) is a genetically determined causal risk factor for myocardial infarction, atherosclerotic stenosis and aortic valve stenosis (reviewed in [2]). Other disease groups, like heart failure and venous thromboembolism, are under current evaluation [2]. Clinical investigation of Lp(a) is largely hampered by the fact that Lp(a)-hyperlipidemia (Lp(a)-HLP) frequently co-incides with other genetic factors for hyperlipidemia and risk of CVD, such as familial hypercholesterolemia (FH) or apolipoprotein E4-allele [3, 4].

Despite extensive research, the underlying mechanisms by which Lp(a) mediates atherogenesis and inflammation, and how Lp(a) contributes to vascular diseases, are still incompletely understood.

## Structure and genetics of Lp(a)

The Lp(a) is largely similar to the low density lipoprotein (LDL) particle, containing one molecule of apolipoprotein B‑100 (apoB-100) around a lipid core of cholesteryl esters, triacylglycerols, diverse phospholipids and unesterified cholesterol. In addition, a highly glycosylated, hydrophilic apolipoprotein (a) (apo(a)) is unique for Lp(a), distinguishing Lp(a) from LDL (reviewed in [5]). Apo(a) shows a high amino acid sequence homology to the serine protease plasminogen, and consists of a carboxy-terminal, enzymatically inactive, protease-like domain followed by a kringle-V domain and a variable kringle-IV (K-IV) domain ([5] and references therein). The K‑IV domain of apo(a) appears as 10 diverse types (T1-T10) [5]. While T1 and T3-T10 are present as a single copy in one apo(a) molecule, the number of T2 varies between 3 and 43 ([5] and references therein). As a consequence, the different repeats of K‑IV_T2_ in apo(a) account for the high size polymorphism of this apolipoprotein [5]. There is an inverse relationship between the number of K‑IV_T2_ repeats of apo(a) and the level of Lp(a) in plasma ([1, 5] and references therein). The size length of apo(a) largely determines its rate of hepatic synthesis and secretion, and also the mass of circulating Lp(a) [6].

Apo(a) is covalently linked to apoB-100 by a single disulfide bond on K‑IV_T9_, in close proximity to the LDL receptor binding site of apoB-100 (reviewed in [1]). The characteristic ‘lysine binding site’ (LBS) of apo(a), which is critically important for many cellular effects and atherogenic potential of Lp(a), is located on K‑IV_T10_ [1].

Apo(a) is encoded by two alleles of the LPA gene (chromosome 6q26), and several genetic LPA-variants have been published, including variations in the K‑IV repeats, diverse single nucleotide polymorphisms (SNPs) and other promoter variants in the LPA gene region [1, 2, 5]. The plasma concentrations of Lp(a) show significant diversity in ethnical groups (e. g. Caucasians have lower plasma Lp(a) levels than African-Americans), and in individuals carrying apo(a) even of the same size polymorphism [7, 8]. Furthermore, elderly individuals (above 75 yrs) tend to show marginally, but not significantly, elevated plasma levels of Lp(a) as compared to the general population under 75 yrs [9].

## Measurement of Lp(a) in plasma

Although Lp(a) quantification in human plasma (or serum) belongs to standard assays in most clinical laboratories, the precise quantitative analysis of Lp(a) is still a challenge with many pitfalls: (i) Lp(a) particles are present in different molecular masses, depending on the differences in isoform size of apo(a), as it varies between 275 and 800 kDa [10, 11]. Novel commercial Lp(a) assays are now available that are insensitive to the variability in Lp(a) mass isoforms [5]. Lp(a) mass refers to the entire mass of the whole particle, including lipids, proteins and carbohydrates [5, 12]. In addition to the apo(a) isoform mass, however, there is also a large variability in the lipid mass within Lp(a) particles [12]. Thus, availability of particle-based, mass-insensitive Lp(a) assays is of great importance. In a recent paper Guadagno and co-workers presented the validation data of an Lp(a) particle concentration assay by quantitative lipoprotein immunofixation electrophoresis, showing precision and linearity across a 16-fold range, which is suitable for use in clinical laboratories [13]. (ii) Most Lp(a) assays are based on immunological methods (e. g. immunonephelometry, immunoturbidimetry, ELISA) using antibodies against apo(a). In contrast to previous immunoassays with antibodies binding to K‑IV_T2_, antibodies in more recent assays should recognize K‑IV_T9_, since this binding site is more stable than K‑IV_T2_ [10]. (iii) Apo(a) isoforms, selected for previous immunological Lp(a) assay calibrators, are with small apo(a) sizes, thus leading to a consequent overestimation of Lp(a) levels in all clinical samples, in which the apo(a) isoforms are larger than in the calibrator [5]. The approaches for Lp(a) immunoassay optimization by implementation of international reference reagents have partially solved this analytical problem, and some recently available commercial Lp(a) assays provide acceptable robustness and analytical precision ([5] and references therein). In addition, the simultaneous quantitation and size characterization of apo(a) by ultra-performance liquid chromatography/mass spectrometry was also reported [14]. (iv) Lp(a) levels, determined by optimized apo(a) isoform dependent assays (see previous point), are delivered in nmol/L [5], and this form should be preferred in current studies. However, other Lp(a) assays may report their results in mg/L. No factor should be used for conversion of Lp(a) concentrations from mg/L to nmol/L, or *vice versa*, as this may lead to misinterpretation of data and confusion [5]. (v) Currently, there is no generally accepted patient threshold for elevated plasma Lp(a) and the prevalence of Lp(a)-HLP in the general population is not yet defined. In a very recent study an attempt has been made to assess the distribution of plasma Lp(a) in a large US-cohort, and the data may influence consensus documents, guidelines and therapeutic cut-offs for Lp(a)-mediated risk for CVD in the future [15].

## Function of Lp(a)

The physiological role of Lp(a) in human is still not fully elucidated, and individuals with extremely low levels of plasma Lp(a) present no disease or deficiency syndromes [6]. Earlier works agree that Lp(a) accelerates wound healing and tissue repair, and therefore Lp(a) provided an evolutionary advantage to humans [6, 16]. Indeed, Lp(a) accumulates in endothelial injuries, binds to several components of the vessel wall and the sub-endothelial matrix, stimulates chemotactic activation of monocytes/macrophages, modulates angiogenesis, and all these effects are mediated by apo(a) [6, 16].

### Effects of Lp(a) on vascular endothelial cells

In previous years Koschinsky and co-workers have demonstrated in consecutive papers how apo(a) modifies the cellular function of cultured vascular endothelial cells: (i) Apo(a) stimulates migration and proliferation of HUVECs (human umbilical-vein endothelial cells) through a mechanism involving CD51/CD61 (integrin α_V_β_3_), Src and MAP (mitogen-activated protein) kinases, including ERK (extracellular-signal-regulated kinase), p38 and JNK (c-Jun *N*-terminal kinase) [17]. (ii) Apo(a) induces a RhoA/Rho-kinase signalling, leading to formation of stress fibers, endothelial contraction and vascular permeability [18]. (iii) Apo(a) disrupts VE-cadherin/β-catenin complexes via a Src-dependent mechanism, which leads to decreased phosphorylation of β‑catenin, parallel with increased phosphorylation of Akt and glycogen synthase kinase-3β. This phosphorylation-shift leads to an increased nuclear translocation of β‑catenin and subsequent induction of cyclooxygenase-2 (COX-2) gene expression, thus mediating enhanced prostaglandin E2 (PGE_2_) formation [19]. (iv) Apo(a) increases the gene expression of certain adhesion molecules, including CD54 (ICAM-1) and CD62E (E-selectin) by a yet unclear mechanism ([20] and references therein). (v) Apo(a) inhibits pericellular activation of plasminogen by a mechanism involving urokinase-type plasminogen activator (uPA) and CD87 (uPA-receptor), resulting in decreased myoepithelial organization and formation of new vascular tubes ([20] and references therein).

### Effects of Lp(a) on smooth muscle cells

In addition to endothelial cells, Lp(a) also influences the function of smooth muscle cells (SMCs) through apo(a): (i) Apo(a) induces a concentration-dependent chemorepulsion of SMCs in migration assays via a mechanism involving CD51/CD61 and RhoA/Rho-kinase, but not transforming growth factor-β (TGFβ) [21]. (ii) Lp(a) dampens the activity of TGFβ in SMCs, and the factors contributing to this effect have yet to be identified [16].

### Effects of Lp(a) on monocytes/macrophages

It has been demonstrated that Lp(a) promotes the differentiation of pro-inflammatory, M1-type macrophages, that secrete a set of pro-inflammatory cytokines (e. g. interleukin (IL)-1β, IL-6, IL-8, tumor necrosis factor (TNF)-α) and chemokines, like ‘interferon-gamma-induced protein-10’ (IP10, CXCL10) and ‘Regulated on Activation, Normal T‑cell Expressed and Secreted’ (RANTES, CCL5), leading to activation of T‑helper-1 (Th1) cells and natural killer (NK) cells [22–25]. The chemokines CXCL10 and RANTES are accompanied with inhibition of angiogenesis, which may affect function of vasa vasorum [25].

Taken together, the above mentioned cellular effects of Lp(a) underline its function as potent modulator of tissue remodelling, nevertheless the same mechanisms also contribute to sustained plaque development in atherosclerosis.

## Lp(a) and inflammation and atherogenesis

**Fig. 1:**
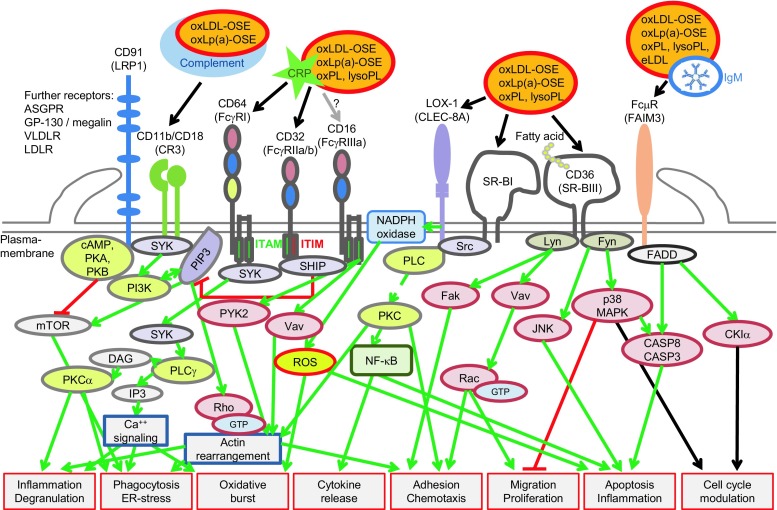
Schematic figure depicting major charge-/motif- and opsonin receptors involved in signaling and clearance of oxidized phospholipids, lysophospholipids, oxidized LDL and Lp(a)

Similarly to other lipoproteins Lp(a) is also susceptible to oxidative modifications, leading to formation of pro-inflammatory and pro-atherogenic oxidized phospholipids, oxysterols, oxidized lipid-protein adducts, termed ‘oxidation-specific epitopes’ (OSEs), produced in response to reactive oxygen species (ROS) ([1, 26] and references therein). Different OSEs are present on Lp(a) as ‘danger-associated molecular patterns’ (DAMPs) that perpetuate local inflammation, apoptosis and tissue disintegration, and they are recognized by a set of pattern-recognition receptors (PRRs) that trigger innate immunity [1, 26]. The most important Lp(a)-associated DAMPs, PRRs and their major downstream signalling mechanisms are summarized in Fig. 1.

By using OSE-specific monoclonal antibodies (particularly T15/E06 IgM), recognizing oxidatively modified but not native phospholipids, Witztum, Tsimikas and colleagues found correlations between plasma levels of oxidized phospholipids and plasma Lp(a), apo(a) size polymorphism, CVD and progression of aortic valve stenosis [1, 26, 27]. Furthermore, by the E06 antibody they were able to block the recognition and uptake of oxidized phospholipids and apoptotic cells by DAMPs, and thus reduce the progression of CVD in atherosclerosis-susceptible mice (reviewed in [1, 26]).

In a recent study Scipione *et al.* have shown that recombinant apo(a) with 17 subdomains within K‑IV induces a dose-dependent increase in expression of IL-8 (mRNA and protein) in macrophage cell lines via CD36 and Toll-like receptor-2 (TLR2)-mediated signalling involving MAP kinases, JNK and ERK [28]. Furthermore, this stimulatory effect was linked to the LBS in K‑IV_T10_ of apo(a), since it was unaffected by trypsin-treatment, but dampened by mutation of the LBS resulting in the loss of oxidized phospholipid on apo(a) [28]. Enzymatic removal of oxidized phospholipid from apo(a) also blunted the IL-8 inducing effect of apo(a), thus verifying the LBS as OSE [28]. Consistent with these results the same group also showed increased monocyte trafficking to the arterial wall in subjects with Lp(a)-HLP, as compared to probands with normal Lp(a) levels, using *in vivo* high resolution imaging techniques [29]. In addition, a parallel ex vivo approach demonstrated the pro-inflammatory activation of monocytes in Lp(a)-HLP [29]. Furthermore, *in vitro* experiments revealed that Lp(a) from Lp(a)-HLP patients contained oxidized phospholipids, and this modified Lp(a) augmented the pro-inflammatory response of monocytes derived from healthy control subjects [29]. The effect was markedly attenuated by inactivating oxidized phospholipids on Lp(a) or removing them from apo(a) [29]. In this scenario not apo(a) itself, but rather OSEs in apo(a) are responsible for the (patho)physiological function(s) of Lp(a), and the LBS is one of the important OSEs in Lp(a).

In addition, modified Lp(a) binds and carries pro-inflammatory molecules such as the monocyte chemoattractant protein-1 (MCP-1/CCL2), a major chemokine inducing and maintaining vascular inflammation, and oxidized phospholipids of modified Lp(a) were shown to be major determinants for MCP-1 binding [30].

Taken together, Lp(a) promotes inflammation and atherosclerosis by multiple mechanisms, and OSEs of apo(a) are critically involved in the pathogenesis of Lp(a) (Fig. 1).

Association studies have shown that plasma levels of IgM antibodies against OSEs are inversely correlated with CVD (reviewed in [31]), and OSEs are major targets of pre-immune, natural IgM antibodies in humans and mice [32]. Approximately 30% of all natural IgM antibodies, recognizing diverse OSEs, were found to bind to atherosclerotic lesions and apoptotic cells in a mouse model [32]. Furthermore, genetic deficiency of secreted IgM (*sIgM* ^-/-^) in mice dramatically accelerates atherosclerosis on the LDL receptor deficient (*Ldlr* ^-/-^), atherosclerosis-susceptible mouse background, parallel with increased plasma IgE levels and perivascular accumulation of mast cells and neutrophils within the plaques [33]. In addition, the OSE-specific T15/E06 IgM blocks the recognition of the oxidized phospholipid POVPC (1-palmitoyl-2-(5-oxovaleroyl)-*sn*-glycero-3-phosphorylcholine) by the scavenger receptor CD36 [34], and the IL-6 secretion, induced by oxPAPC (oxidized 1‑palmitoyl-2-arachidonoyl-*sn*-glycero-3-phosphocholine) in macrophages [35]. The OSE-specific natural NA17 IgM, recognizing a malondialdehyde (MDA) epitope, binds apoptotic cells and promotes their clearance by macrophages [32]. These findings clearly demonstrate the atheroprotective role of natural, OSE-specific IgM antibodies [31].

Autoantibodies against modified LDL were demonstrated in human plasma, and increased levels of these autoantibodies correlated with severe atherosclerosis [36]. Interestingly, elevated levels of IgM autoantibodies against OSE-specific apoB-immune complexes reduced the cardiovascular risk of Lp(a) in a recent epidemiological study [37]. In previous works Romero *et al.* reported autoantibodies against MDA-modified Lp(a) in plasma of patients with antiphospholipid syndrome (APS) [38] and systemic lupus erythematosus (SLE) [39], thus linking Lp(a) to autoimmunity. Indeed, similarly to the formation of OSE-specific IgM autoantibodies against modified LDL, autoantibodies against oxidatively modified epitopes of Lp(a) can also be developed, and these autoantibodies may attenuate the pro-inflammatory and pro-atherogenic signalling of Lp(a).

## Lp(a) and malignancies

It was demonstrated nearly two decades ago that angiostatin, a cancer-mediated proteolytic fragment of plasminogen, is able to inhibit metastasis and neovascularization of tumors [40]. Based on the high grade of homology between plasminogen and apo(a), the potential anti-neoplastic role of Lp(a) was always postulated, but convincing evidence has not yet been demonstrated ([6] and references therein).

Taking into account that Lp(a) shows growth-factor-like properties, including stimulation of angiogenesis, one would rather expect that Lp(a) promotes tumor growth, as malignant cells would likely benefit from neovascularization. Regarding that Lp(a)-HLP is associated with increased amounts of oxidized phospholipids and OSEs, it is tempting to speculate that autoantibodies against oxidized phospholipids develop in Lp(a)-HLP, and these antibodies may antagonize some OSE-associated pro-inflammatory and angiogenic events. Finally, the dampened angiogenic signalling may thus reduce vascularization of tumors.

Previous studies in animal models suggest that the proteolytic break-down fragments of apo(a) are responsible for the anti-angiogenic and anti-neoplastic function of Lp(a) (reviewed in [41]). In a very recent study Lp(a) and vitamin C were found to impair development of breast cancer in a mouse model, transgenic for human Lp(a) and deficient in endogenous vitamin C production [42].

Despite promising *in vivo* and *in vitro* results epidemiological studies have failed to clearly link Lp(a) with tumor protection so far. In a recent work Sawabe and colleagues reported a link between low Lp(a) levels and all-cause and cancer death [43]. The same group found Lp(a)-HLP as a significant risk factor for any origins of cancer [44]. Furthermore, increased Lp(a) levels in plasma is commonly reported in tumor patients in comparison to controls, irrespective of source of the tumor [6, 41, 45, 46].

Taken together, the current knowledge about the anti-neoplastic function is insufficient and further investigations are required to clarify this important question.

## Conclusion

In recent years several important consecutive data have been presented showing cellular and molecular mechanisms of Lp(a) action. Convincing evidence has been presented that the pro-inflammatory and pro-atherosclerotic effects of Lp(a) are largely attributed to different OSEs present in Lp(a) particles.

Different OSEs are major targets of pre-immune, natural IgM antibodies, and autoantibodies against oxidized phospholipids may develop in Lp(a)-HLP, thus antagonizing the detrimental effects of Lp(a).

The anti-neoplastic effect of Lp(a) is not convincingly demonstrated, and further investigations are necessary to clarify this question.

Despite emerging data from clinical and experimental studies our current knowledge about the impact of Lp(a)-HLP in diverse pathologies is still rudimentary. For further studies establishment of novel biomarkers and particle-based, mass-insensitive assays for quantification of Lp(a) are essentially required.
